# 1591. Real-world experiences and outcomes implementing long-acting cabotegravir/rilpivirine at a Ryan White HIV/AIDS Program (RWHAP)-funded clinic in South Florida

**DOI:** 10.1093/ofid/ofad500.1426

**Published:** 2023-11-27

**Authors:** Sheila Montalvo, Elizabeth Sherman, Paula A Eckardt, Romina Bromberg, Kenneth K Poon, Garrett Van Ostran, Edison Cano Cevallos, Angela Savage

**Affiliations:** Memorial Hospital System, Cooper City, Florida; Memorial Healthcare Systems, Hollywood, Florida; Memorial Healthcare System, Hollywood, Florida; Memorial Healthcare Systems, Hollywood, Florida; Memorial Healthcare System, Hollywood, Florida; Memorial Healthcare Systems, Hollywood, Florida; Memorial Healthcare System, Hollywood, Florida; Memorial Healthcare Systems, Hollywood, Florida

## Abstract

**Background:**

Long-acting (LA) cabotegravir/rilpivirine (CAB/RPV) may simplify antiretroviral therapy (ART), improve adherence, and reduce pill stigma for people with HIV (PWH). However, real-world implementation is challenged by various barriers. We describe an integrated LA CAB/RPV workflow and the clinical characteristics and outcomes of PWH referred for and initiated on CAB/RPV in a Ryan White HIV/AIDS Program-funded clinic in South Florida (serving more than 1600 PWH).

**Methods:**

The clinic engaged an interdisciplinary team (MD/APRN, PharmDs, RNs) to develop and maintain an infrastructure required to transition virologically suppressed (HIV RNA < 50 copies/mL) PWH from oral ART to LA CAB/RPV (Figure 1). MD/APRN referred pre-screened and interested PWH to PharmDs for eligibility evaluation, medication counseling, and drug acquisition. RNs administered injections, scheduled and tracked clinic appointments to ensure on-time injections, and ordered appropriate labs. PWH followed up with MD/APRN every 4 months to ensure efficacy and safety.

**Figure:**
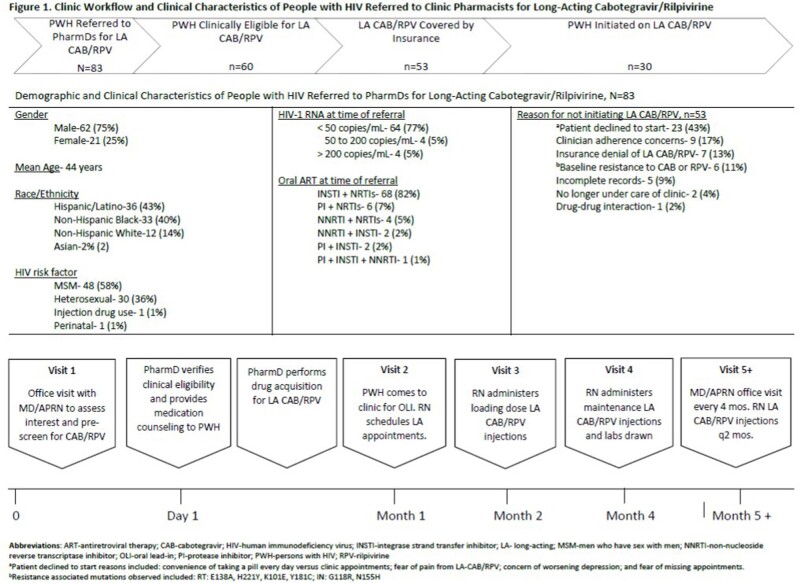

**Results:**

Between January 2022 and March 2023, 83 PWH were referred to PharmDs for initiation of LA CAB/RPV and 30 (36%) initiated LA CAB/RPV. Those not initiated on LA CAB/RPV included those who declined to start (e.g., patient concerns over side effects, convenience) (n=23), clinician adherence concerns (n=9), insurance denial (n=7), and baseline resistance (n=7). Payor source for LA CAB/RPV included 40% RWHAP, 36% private, and 24% Medicare/Medicaid. For those initiated on LA CAB/RPV (Table 1), we observed a mean turnaround time of 23 days from PharmD eligibility evaluation to the first injections. Three PWH discontinued LA CAB/RPV: 2 due to side effects and 1 due to pregnancy. Five PWH experienced low-level viremia (HIV RNA < 200 copies/mL) following the switch, all others maintained viral suppression.

**Figure:**
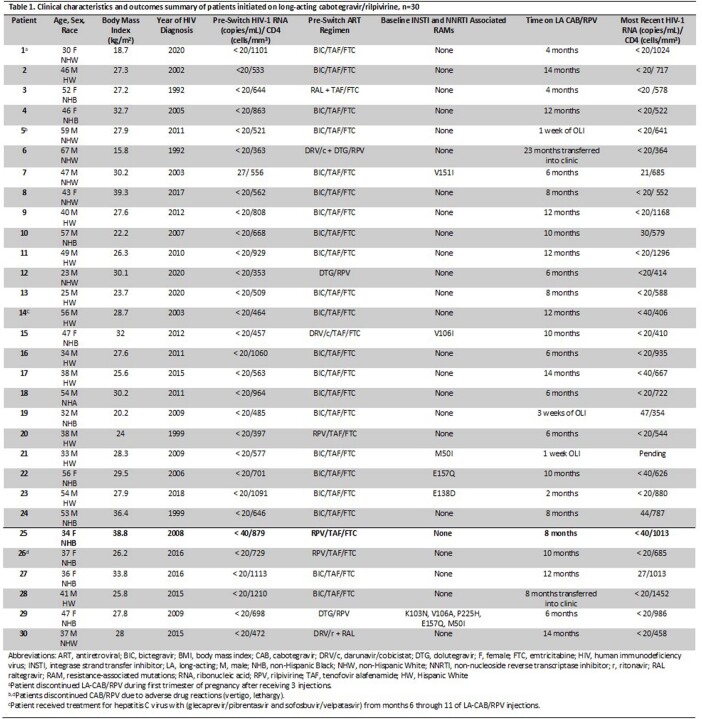

**Conclusion:**

A large number of eligible and interested PWH referred for LA CAB/RPV did not initiate the drug. Those who initiated CAB/RPV tolerated the drug well and maintained viral suppression. Implementation of LA CAB/RPV is enhanced by an interdisciplinary team to provide services, optimize workflows, and maintain infrastructure needed for a successful program.

**Disclosures:**

**All Authors**: No reported disclosures

